# Single-stage multidisciplinary management of contiguous infection involving infected abdominal aortic aneurysm and lumbar spondylodiscitis following infective endocarditis

**DOI:** 10.1016/j.jvscit.2026.102351

**Published:** 2026-06-09

**Authors:** Jun Iida, Hisao Harada, Ryoko Arakaki, Tetsuji Inoue, Seiya Saito, Mayuko Ouchi, Yoshio Arai

**Affiliations:** a Department of Cardiovascular Surgery, Kumamoto Chuo Hospital, Kumamoto, Japan; b Department of Orthopedic Surgery, Kumamoto Chuo Hospital, Kumamoto, Japan; c Department of Gastrointestinal Surgery, Kumamoto Chuo Hospital, Kumamoto, Japan

**Keywords:** Infected abdominal aortic aneurysm, Spondylodiscitis, Infective endocarditis, Multidisciplinary surgery, In situ reconstruction

## Abstract

Infective endocarditis can cause septic embolization and metastatic infections, including infected aortic aneurysm and spondylodiscitis. We report the case of a 77-year-old man with contiguous infection who was successfully treated using a single-stage multidisciplinary approach. Following antibiotic therapy that controlled the endocarditis, rapid expansion of an abdominal aortic aneurysm indicated impending rupture. Simultaneous surgery included in situ reconstruction using a rifampicin-soaked graft, infection-avoiding graft routing, spinal debridement, autologous bone reconstruction, and omental coverage. The patient recovered without recurrent infection. This case suggests that a single-stage multidisciplinary approach combining multiple surgical strategies may be an effective option for managing complex contiguous infections.

Infective endocarditis (IE) remains a serious disease associated with high morbidity and mortality due to septic embolization and metastatic infections. Infected aortic aneurysm and pyogenic spondylodiscitis are rare but life-threatening complications, and their coexistence presents significant challenges in determining the optimal treatment strategies. Although staged procedures may reduce surgical invasiveness, urgent intervention is often required in the cases of impending aneurysm rupture. Furthermore, leaving adjacent infectious foci untreated may increase the risk of reinfection following vascular reconstruction.

We report a case of an infected abdominal aortic aneurysm (AAA) and lumbar spondylodiscitis secondary to IE that was successfully managed with a single-stage multidisciplinary surgical approach. The patient signed informed consent for publication of this case report.

## Case presentation

A 77-year-old man with a history of distal gastrectomy with Roux-en-Y reconstruction for gastric cancer presented with fever, general fatigue, and dysarthria. Brain magnetic resonance imaging revealed acute cerebral infarction. Blood cultures grew *Streptococcus oralis*, and echocardiography demonstrated vegetations on the mitral valve, leading to the diagnosis of IE.

Because the vegetation showed low mobility and mitral regurgitation was not severe, conservative treatment with intravenous antibiotic therapy was initiated. Approximately 1 month later, the patient developed lower back pain, and lumbar magnetic resonance imaging revealed pyogenic spondylodiscitis (Fig 1).

**Fig 1:**
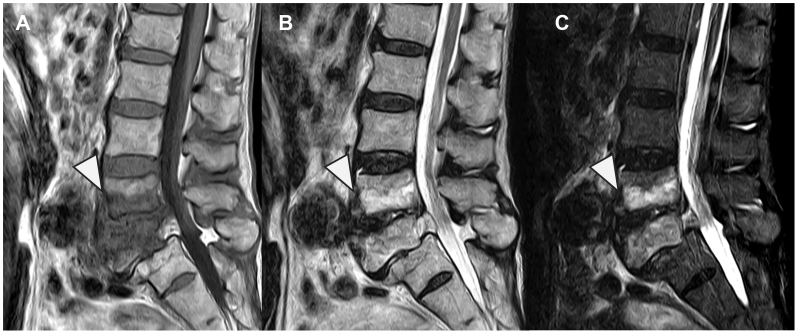
Magnetic resonance imaging findings of lumbar pyogenic spondylodiscitis. **A,** T1-weighted sagittal image showing low-signal intensity in the vertebral body and intervertebral disc (*white arrowheads* indicate the same abscess in all panels). **B,** T2-weighted sagittal image showing high-signal intensity in the same region (*white arrowheads*). **C,** T2-weighted short tau inversion recovery sagittal image demonstrating hyperintensity (*white arrowheads*).

Concurrently, rapid morphological changes were observed in a previously diagnosed AAA, leading to the diagnosis of infected AAA with impending rupture (Fig 2). Inflammatory markers had normalized, and blood cultures had become negative, indicating clinical control of IE.

**Fig 2:**
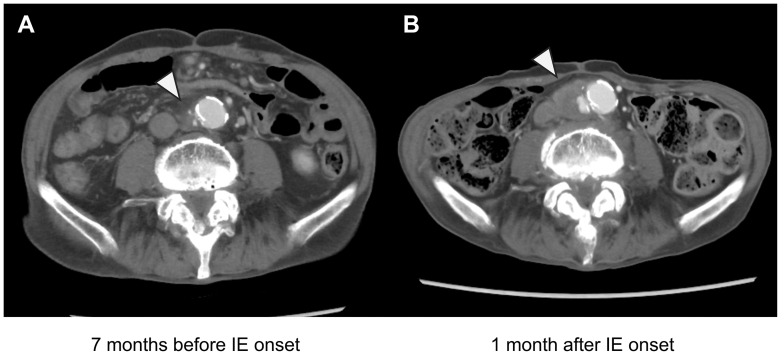
Contrast-enhanced computed tomography findings of an infected abdominal aortic aneurysm. **A,** A pre-existing abdominal aortic aneurysm is observed 7 months before the onset of infective endocarditis (*IE*) (*white arrowhead*). **B,** Rapid aneurysmal expansion with morphologic changes is observed 1 month after onset (*white arrowhead*). The aneurysmal segment is outlined with a *red dashed line* in both panels.

Semiurgent surgical intervention for the infected AAA was required. However, the adjacent untreated spinal infection was considered to pose a high risk of graft reinfection. Therefore, a single-stage multidisciplinary surgical approach was planned.

Because the patient had previously undergone robot-assisted distal gastrectomy with Roux-en-Y reconstruction, the availability of the greater omentum for graft coverage was uncertain. Therefore, alternative reconstructive options, including a rectus abdominis muscle flap, were considered preoperatively.

During adhesiolysis, a viable residual omentum was identified and preserved for graft coverage.

The vascular surgery team performed complete resection of infected aortic tissue followed by in situ reconstruction using a rifampicin-soaked prosthetic graft. The graft was routed through a pathway separate from the infected spinal field, representing an infection-avoiding graft-routing strategy (Fig 3).

**Fig 3:**
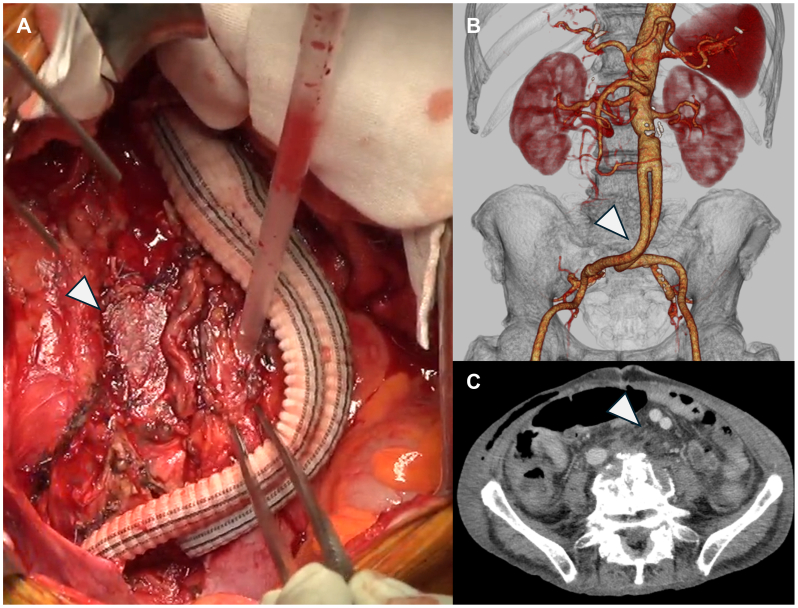
Infection-avoiding graft routing demonstrated by intraoperative findings and postoperative computed tomography. **A,** The graft is routed away from the infected spinal field. The *white arrowhead* indicates the infected field anterior to the vertebral body exposed after aortic wall resection. **B,** Postoperative contrast-enhanced computed tomography confirms preservation of the graft configuration (*white arrowhead*). **C,** The graft is separated from the infected spinal field by an omental flap (*white arrowhead*).

After extensive irrigation, the orthopedic surgery team performed L4/5 discectomy, curettage, and resection of the necrotic vertebral end plate, followed by anterior spinal reconstruction using an autologous iliac crest bone graft without instrumentation (Fig 4). The anterior vertebral periosteum was closed to the extent feasible to separate the spinal field from the vascular graft. The graft was repeatedly irrigated intraoperatively and finally covered with the omental flap.

**Fig 4:**
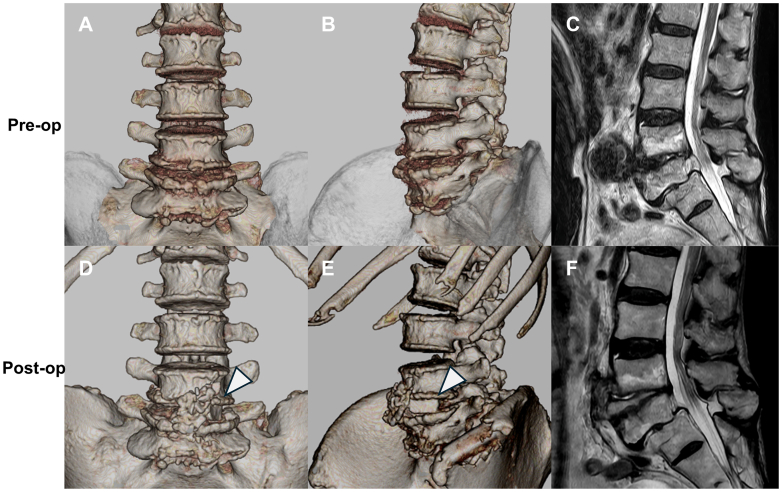
Spinal reconstruction without instrumentation. **A,** Preoperative anterior view of lumbar three-dimensional computed tomography (3D-CT). **B,** Preoperative left anterolateral view of lumbar 3D-CT. **C,** Preoperative T2-weighted sagittal magnetic resonance imaging (MRI). **D,** Postoperative anterior view of lumbar 3D-CT. The *white arrowhead* indicates anterior fixation at L4/5 using an autologous bone graft. **E,** Postoperative left anterolateral view of lumbar 3D-CT. The *white arrowhead* indicates anterior fixation at L4/5 using an autologous bone graft. **F,** Postoperative T2-weighted sagittal MRI. No new vertebral malalignment is observed postoperatively, and anterior lumbar fixation is achieved without instrumentation.

Intraoperative cultures from the aneurysm, vertebral body, and intervertebral disc showed no growth of pathogenic organisms.

Postoperatively, the patient developed chylous ascites, which subsequently resolved with temporary abdominal drainage and conservative management. No recurrence of infection was observed, and the patient was discharged approximately 3 months after surgery with continued oral antibiotic therapy (Fig 5).

**Fig 5:**
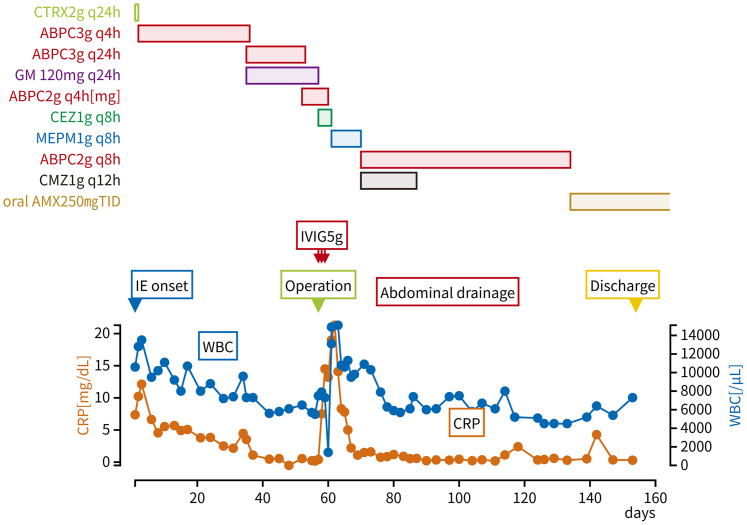
Clinical course, including inflammatory markers and antimicrobial therapy. C-reactive protein (*CRP*) and white blood cell (*WBC*) levels are shown as line graphs, and antimicrobial therapy is presented as horizontal bars. IE, infective endocarditis.

## Discussion

IE can cause metastatic infections through septic embolization, particularly in cases involving viridans group streptococci.^1^^,^^2^ In this case, hematogenous dissemination likely resulted in both an infected AAA and lumbar pyogenic spondylodiscitis. The vertebral end plates are highly vascularized and susceptible to bacterial seeding,^3^^,^^4^ whereas the aneurysmal wall can undergo rapid destruction once infected.

This case demonstrates clinically significant contiguous infection involving the heart, aorta, and spine. Such complex presentations are rare and require careful integration of surgical strategies.

The most critical decision was whether to perform staged or single-stage surgery. Staged procedures may reduce operative stress but carry a risk of persistent infection and graft contamination.^5^^,^^6^ In contrast, single-stage intervention enables simultaneous source control of contiguous infection, potentially reducing the risk of reinfection.^7^ In this case, surgical intervention was performed after achieving adequate infection control of IE, which likely contributed to the favorable outcome.

Treatment strategy selection also required consideration of open vs endovascular repair. Open surgery allowed complete removal of infected tissue and definitive reconstruction. Endovascular repair was not selected in this case because adequate debridement could not be achieved; however, it may be a viable option in selected high-risk patients.^8^

Multiple adjunctive strategies were combined to minimize the risk of reinfection. These included the use of a rifampicin-soaked graft, infection-avoiding graft routing, spinal reconstruction without instrumentation, and omental coverage. This integrated approach combined the advantages of in situ reconstruction with infection avoidance.

Extra-anatomic bypass has traditionally been used to reduce the risk of infection and may be particularly useful in cases with uncontrolled infection, persistent bacteremia, gross purulence, or extensive contamination of the operative field.^9^ However, this approach is associated with lower long-term patency and risks such as stump-related complications.^10^^,^^11^ In contrast, in situ reconstruction has demonstrated favorable outcomes in recent reports.^10^^,^^12^^,^^13^ In the present case, inflammatory markers had normalized, blood cultures were negative, and no gross purulence was identified intraoperatively. Furthermore, the infection-avoiding graft-routing strategy, combined with omental flap coverage, further enhanced infection control by physically separating the graft from the infected spinal field.

Because the extent of omental preservation after previous gastrectomy could not be confirmed preoperatively, alternative reconstructive strategies, including muscle-flap reconstruction, were considered.^14^

This report has several limitations. First, it describes a single case, limiting generalizability. Second, long-term follow-up is limited, and the risk of late graft infection cannot be completely excluded. Third, the durability of spinal reconstruction without instrumentation remains uncertain. Finally, the individual contribution of each component of the combined strategy cannot be determined.

Despite these limitations, this case suggests that a carefully planned single-stage multidisciplinary approach may provide effective source control and durable reconstruction in the selected patients with complex contiguous infections.

## Conclusions

A single-stage multidisciplinary approach performed after achieving infection control of IE with antibiotic therapy enabled successful management of an infected AAA with concomitant lumbar pyogenic spondylodiscitis following IE. This approach combined in situ reconstruction using a rifampicin-soaked graft, infection-avoiding graft routing, spinal reconstruction without instrumentation, and omental coverage.

Careful timing of surgery and close multidisciplinary collaboration are essential in managing such complex infectious conditions.

## Funding

None.

## Disclosures

None.
